# Development of a High‐Resolution Melting (HRM)‐Based Multiplex Real‐Time PCR Assay of PEDV, TGEV, PoRV, PDCoV, PRV, and PRRSV

**DOI:** 10.1155/tbed/1574555

**Published:** 2026-06-30

**Authors:** Fangshan Wang, Jing Ren, Yawen Wang, Chen Yuan, Tairan Sun, Jinhai Huang, Qinye Song

**Affiliations:** ^1^ College of Veterinary Medicine, Hebei Agricultural University, Baoding, 071000, Hebei, China, hebau.edu.cn; ^2^ Hebei Province Institute of Animal Husbandry and Veterinary Medicine, Baoding, 071000, Hebei, China; ^3^ Veterinary Biological Technology Innovation Center of Hebei Province, Baoding, 071000, China; ^4^ Baoding Animal Disease Control and Prevention Center, Baoding, 071000, Hebei, China; ^5^ School of Life Sciences, Faculty of Medicine, Tianjin University, Tianjin, 300072, China, tju.edu.cn

**Keywords:** high-resolution melting (HRM), major porcine viral pathogens, multiplex real-time PCR, simultaneous and differential detection

## Abstract

Six key viral pathogens—porcine epidemic diarrhea virus (PEDV), transmissible gastroenteritis virus (TGEV), porcine rotavirus (PoRV), porcine deltacoronavirus (PDCoV), pseudorabies virus (PRV), and porcine reproductive and respiratory syndrome virus (PRRSV)—inflict substantial economic losses on the global swine industry. Their frequent cocirculation and the resulting mixed infections severely complicate rapid and accurate diagnosis. To overcome this challenge, we developed a multiplex real‐time PCR assay integrated with high‐resolution melting (HRM) analysis for their simultaneous detection and differentiation. Specific primer sets were designed and optimized to target conserved regions of the PEDV‐M, TGEV‐N, PoRV‐VP6, PDCoV‐M, PRV‐gE, and PRRSV‐ORF5 genes. Reaction conditions and HRM parameters were refined, ensuring specific amplification and distinct melting profiles for all six viruses. The assay exhibited excellent specificity, showing no cross‐reactivity with other common porcine pathogens. Limits of detection (LODs) were established at 10 copies/μL for five viruses (PEDV, TGEV, PoRV, PDCoV, and PRRSV) and 100 copies/μL for PRV. Amplification efficiency ranged from 90% to 102% (*R* ^2^ ≥ 0.98). High reproducibility was confirmed by intra‐ and interassay coefficients of variation (CVs) of <3% for cycle threshold (*C*_*t*_) values and <0.5% for melting temperature (*T*_*m*_) values. Clinical validation of 248 field samples demonstrated that detection rates for the six pathogens were 16.0%−27.3% higher (an average of 18.1%) than those of conventional PCR assays. Collectively, these findings confirm that the HRM‐based multiplex real‐time PCR assay is a rapid, sensitive, specific, and highly reproducible tool for detecting and differentiating major porcine viral pathogens. This methodology offers a valuable molecular diagnostic platform, supporting early diagnosis, epidemiological surveillance, and enhanced control of swine viral diseases.

## 1. Introduction

Viral pathogens, including porcine epidemic diarrhea virus (PEDV), transmissible gastroenteritis virus (TGEV), porcine rotavirus (PoRV), porcine deltacoronavirus (PDCoV), pseudorabies virus (PRV), and porcine reproductive and respiratory syndrome virus (PRRSV), collectively pose a significant threat to the global swine industry [1–3]. PEDV and TGEV cause acute viral gastroenteritis with high neonatal mortality [4, 5]. PoRV is a major etiological agent of neonatal diarrhea, leading to growth retardation and substantial economic loss [6]. PDCoV, an emerging enteric coronavirus, induces severe diarrhea and demonstrates potential cross‐species transmissibility [7]. PRV causes pseudorabies, resulting in reproductive failure in sows and neurological disorders in piglets [8], while PRRSV remains an economically critical pathogen responsible for reproductive failure and respiratory disease across all age groups [9].

The frequent co‐occurrence of these pathogens presents a major impediment to effective disease control [10]. Clinical symptoms are often indistinguishable, making differential diagnosis based on the presentation alone unreliable [11]. Conventional methods, such as virus isolation and immunological assays, are too slow and labor‐intensive for large‐scale or onsite detection [12]. Although single‐target real‐time PCR is highly specific and sensitive, its limited throughput renders it inefficient for mixed‐infection screening [13]. Existing multiplex TaqMan‐based PCR assays are constrained by the number of available fluorescence channels and high reagent costs [14]. Consequently, an urgent requirement exists for a rapid, high‐throughput, and cost‐effective molecular approach capable of simultaneously detecting and differentiating these viruses.

High‐resolution melting (HRM) curve analysis is a post‐PCR technique that distinguishes DNA amplicons based on subtle variations in melting temperature (*T*_*m*_), which reflect differences in GC content, sequence length, and base composition [15]. When combined with quantitative PCR (qPCR), HRM enables real‐time amplification monitoring and postamplification sequence discrimination within a single, closed‐tube reaction [16]. This technique offers high specificity, sensitivity, and inherent cost‐effectiveness as it obviates the need for fluorescent probes or labeled primers [17]. HRM‐based assays have proven successful in pathogen detection, genotyping, and single‐nucleotide polymorphism (SNP) analysis [18–20].

To address the current diagnostic gap, this study developed and systematically optimized a multiplex HRM‐based real‐time PCR assay for the concurrent detection and differentiation of the six aforementioned porcine viral pathogens. The assay’s analytical performance was rigorously evaluated concerning specificity, sensitivity, reproducibility, and clinical applicability. This methodology establishes a powerful and economical diagnostic platform for rapid identification and epidemiological surveillance, thereby strengthening prevention and control strategies within the swine industry.

## 2. Materials and Methods

### 2.1. Sample Collection and Preparation

A total of 248 clinical samples—including blood, saliva, intestinal tissues, intestinal contents, mesenteric lymph nodes, and feces—were collected from 15 large‐scale pig farms located in Shandong, Hunan, Guangxi, and other provinces of China. Reference strains of PEDV, TGEV, PoRV, PDCoV, PRV, and PRRSV, as well as classical swine fever virus (CSFV), porcine circovirus type 2 (PCV2), porcine circovirus type 3 (PCV3), porcine parvovirus (PPV), and Japanese encephalitis virus (JEV), were obtained from Shandong Xinde Technology Co., Ltd. (Shandong, China). The detailed genotype information and GenBank reference accession numbers of all reference strains are listed as follows: PEDV, G2c subtype, AY974335; TGEV, classical epidemic strain, DQ443743; PoRV, G9 genotype, JQ343834.1; PDCoV, Chinese main epidemic strain, MN249445; PRV, variant strain, OR062216.1; PRRSV, Lineage 1 NADC30‐like, MW053401.1; CSFV, genotype 2.1, MH891919.1; PCV2, PCV2d genotype, MK303459.1; PCV3, PCV3a genotype, OQ389701.1; PPV, NADL‐2 strain, NC_001718.1; and JEV, genotype I, MW732041.1. In addition, healthy porcine samples, including intestinal tissues, feces, lung, and mesenteric lymph nodes, were collected for matrix specificity validation.

Viral nucleic acids (DNA/RNA) were extracted from both reference strains and clinical samples via the FinePure Virus DNA/RNA Extraction Kit (Jifan Biotechnology, China) according to the manufacturer’s instructions. For RNA viruses (PEDV, TGEV, PoRV, PDCoV, and PRRSV), complementary DNA (cDNA) was synthesized using the EasyScript One‐Step RT‒PCR SuperMix (TransGen Biotech, China) under the following conditions: 42°C for 30 min and 85°C for 5 s. The extracted DNA and synthesized cDNA were stored at −20°C until further analysis.

### 2.2. Primer Design

Conserved gene sequences of PEDV‐M, TGEV‐N, PoRV‐VP6, PDCoV‐M, PRV‐gE, and PRRSV‐ORF5 were retrieved from the NCBI GenBank database. Multiple sequence alignments were conducted using MEGA 7.0 software to identify conserved regions.

Specific primers were designed using Beacon Designer 8.14 (Premier Biosoft, USA), ensuring that the amplicons exhibited at least a 1°C difference in melting temperature (*T*_*m*_) to allow discrimination in HRM analysis. The specificity of all primers was verified by NCBI BLAST analysis. Primers were synthesized by Sangon Biotech Co., Ltd. (Shanghai, China), and their sequences are listed in Table 1.

**Table 1:** Primer sequences information for multiplex HRM real‐time PCR.

Virus	Gene	Primer sequence (5′–3′)	Reference sequence	Length (bp)
PEDV	M	F: GTGGTACATTGCTTGTAG	AY974335	78
		R: CGACTGTGACGAAATTAG		
TGEV	N	F: GAGCTAGAAGCAGTTCAG	DQ443743	101
		R: CACAGATGGAACACATTC		
PoRV	VP6	F: ATGGCTAGAGAATCACAA	JQ343834.1	191
		R: GATGCTGAATATGGAAGTATA		
PDCoV	M	F: GCGTAATCGTGTGATCTA	MN249445	128
		R: TGGACACAATGAAAACTG		
PRV	gE	F: CTCCTTCGTGATGACGTG	OR062216.1	163
		R: GTCGTAGTAGTCCTCGTG		
PRRSV	ORF5	F: GGGTTTTCTCACGACAAG	MW053401.1	141
		R: CGGATAACAAAGCATACAAA		

### 2.3. Preparation of Plasmid Standards

The target gene fragments of the six viruses were amplified using the designed primers. The PCR products were separated by 2% agarose gel electrophoresis, and the target bands were collected and purified with a DNA Gel Extraction Kit (Takara Biotechnology Co., Ltd., Dalian, China). The purified fragments were ligated into the pMD18‐T vector (Takara Biotechnology Co., Ltd., Dalian, China) and transformed into *Escherichia coli* DH5α competent cells. Positive clones were screened by PCR using M13 universal primers (M13‐F: 5′‐CGCCAGGGTTTTCCCAGTCACGAC‐3′; M13‐R: 5′‐AGCGGATAACAATTTCACACAGGA‐3′) and confirmed by Sanger sequencing. Recombinant plasmids were extracted using E.Z.N.A. Plasmid Mini Kit I (Omega Bio‐Tek, USA) and plasmid concentrations were quantified via a NanoDrop One Microvolume UV–Vis Spectrophotometer (Thermo Fisher Scientific, USA). The plasmids were stored at −20°C for subsequent use.

### 2.4. Optimization of Single‐ and Multiplex HRM Real‐Time PCR Systems

The single HRM real‐time PCR assay was performed in a 20 μL reaction volume containing 10 μL of 2 × TransStart Tip Green qPCR SuperMix (TransGen Biotech, China), 0.4 μL of each primer (10 μM), 2 μL of template DNA/cDNA, and 7.2 μL of RNase‐free water. The thermal cycling program was as follows: 50°C for 30 min (reverse transcription for RNA viruses) and 95°C for 30 s, followed by 40 cycles of 95°C for 35 s and 60°C for 45 s (fluorescence acquisition). The HRM step consisted of 95°C for 60 s, 40°C for 60 s, and a gradual temperature increase from 65 to 97°C at 0.1°C/s, with fluorescence data collected at each increment. The primer concentrations were optimized (0.2, 0.4, or 0.6 μL per primer) to obtain specific amplification and clear melting curve separation.

Based on the optimized single HRM real‐time PCR conditions, the concentrations of each primer pair in the multiplex assay were adjusted to ensure specific amplification and balanced amplification efficiency of all six viruses. The multiplex reaction system (20 μL) contained 10 μL of 2 × TransStar Tip Green gPCR SuperMix, 2 μL of templates (mixed the standard recombinant plasmids containing six various viral genes or clinical sample cDNA), different concentrations of each primer pair (PEDV: 0.4 μL each; TGEV: 0.2 μL each; PoRV: 0.6 μL each; PDCoV: 0.4 μL each; PRV: 0.4 μL each; PRRSV: 0.6 μL each), and 2.6 μL of RNase‐free water. The reaction program was the same as that for the single HRM real‐time PCR. Strict quality control measures were implemented throughout the assay, including parallel extraction negative controls, no‐template controls (NTCs), and positive controls for each run, to ensure the validity of all test results.

### 2.5. Performance Evaluation of the Multiplex HRM Assay

The multiplex HRM assay specificity was evaluated using the recombinant plasmids (10^5^ copies/μL) of each target virus and the genomic DNA or RNA extracted from other common porcine pathogens (CSFV, PCV2, PCV3, PPV, and JEV) as templates and the nucleic acids extracted from healthy porcine tissue and fecal matrix samples. RNase‐free water was used as a negative control.

Tenfold serial dilutions of recombinant plasmids, ranging from 1 × 10^6^ to 1 × 10^0^ copies/μL, were prepared. Each dilution was tested in triplicate to determine the limit of detection (LOD). Standard curves were generated using *C*_*t*_ values from the dilutions of 1 × 10^6^ to 1 × 10^2^ copies/μL. Amplification efficiencies (*E*) and correlation coefficients (*R* ^2^) were calculated from the slope.

Assay reproducibility was assessed using of plasmid templates at three concentrations (1 × 10^6^, 1 × 10^4^, and 1 × 10^2^ copies/μL). Each concentration was tested in triplicate within a single run (intra‐assay) and across three independent runs (interassay). Coefficients of variation (CVs) for *C*_*t*_ and *T*_*m*_ values were calculated to evaluate precision.

### 2.6. Clinical Sample Detection

The 248 clinical samples were analyzed using the developed multiplex HRM real‐time PCR assay and conventional PCR methods (GB/T 18641‐2018 for PRV, GB/T 36871‐2018 for PEDV/TGEV/PoRV, GB/T 18090‐2023 for PRRSV, and DB31/T 1512‐2024 for PDCoV). The detection rates obtained by both methods were compared to evaluate the clinical applicability of the HRM assay. The primers used for detecting PEDV, TGEV, PoRV, PDCoV, PRV, and PRRSV in accordance with the aforementioned national standards are detailed in Table 2. To validate the performance, HRM‐positive clinical samples (*C*_*t*_ < 28) were verified via Sanger sequencing—the diagnostic gold standard. Following amplification with the established HRM primer sets and purification, sequences were aligned against NCBI GenBank references to confirm the pathogen identity and determine the concordance rate between the two methods.

**Table 2:** Primer sequence information for PCR.

Virus	Gene	Primer sequence (5′–3′)	Length (bp)
PEDV	M	F: TTCGGTTCTATTCCCGTTGATG	663
		R: CCCATGAAGCACTTTCTCACTATC	
TGEV	N	F: TTACAAACTCGCTATCGCATGG	528
		R: TCTTGTCACATCACCTTTACCTGC	
PoRV	M	F: ACTTATTCTGCTTTGGCTGCT	504
		R: GAAGTGGTTATGGTGTGAAGTC	
PDCoV	VP7	F: CCCCGGTATTGAATATACCACAGT	333
		R: TTTCTGTTGGCCACCCTTTAGT	
PRV	gE	F: TTTGGATCCATGCGGCCCTTTCTG	368
		R: TTTGAATTCTTACGACACGGCGTCGCA	
PRRSV	ORF7	F: ATGGCCAGCCAGTCAATCA	PRRSV‐1: 398 PRRSV‐2: 443
		R: TCGCCCTAATTGAATAGGTGACT	

## 3. Results

### 3.1. Construction and Identification of the Plasmid Standards

**Figure 1:**
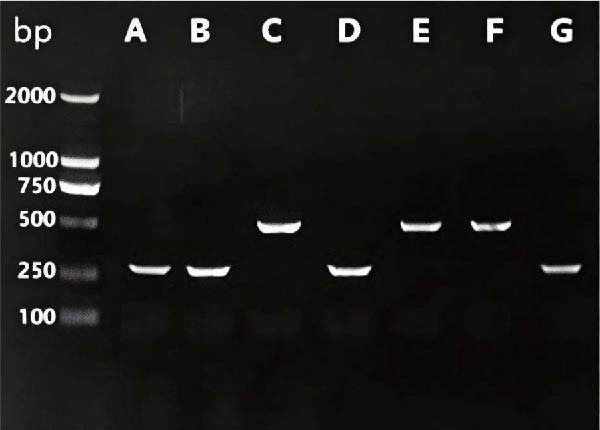
PCR identification of recombinant plasmids. M: 2000 bp DNA Ladder; (A–F) recombinant plasmids of PEDV M gene, TGEV N gene, PoRV VP6, PDCoV M gene, PRV gE, and PRRSV ORF5, respectively; (G) empty pMD18‐T vector negative control.

PCR amplification of the recombinant plasmids generated clear amplicons of the expected sizes (348 bp for PEDV, 371 bp for TGEV, 461 bp for PoRV, 398 bp for PDCoV, 433 bp for PRV, and 411 bp for PRRSV) (Figure 1). Sequencing confirmed that the inserted fragments perfectly matched the target gene sequences. The concentrations of the recombinant plasmids ranged from 53 to 112 ng/μL, and the copy numbers ranged from 1.72 × 10^10^ to 3.59 × 10^10^ copies/μL.

### 3.2. Specificity and *T*_*m*_ Values of Single HRM Real‐Time PCR

**Figure 2:**
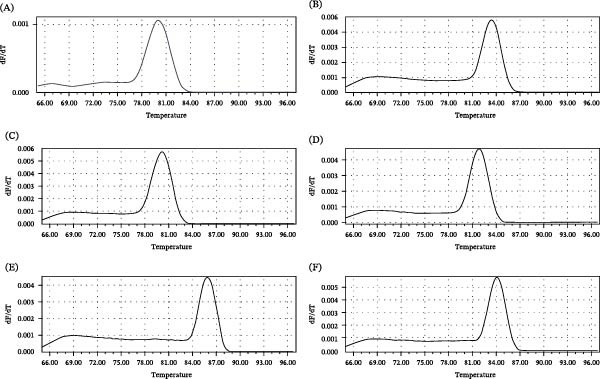
Melting curves of single HRM real‐time PCR assays. (A) PEDV; (B) TGEV; (C) PoRV; (D) PDCoV; (E) PRV; (F) PRRSV.

Each single HRM real‐time PCR assay produced a specific single melting peak, and no primer dimer was observed in the negative control (Figure 2). The *T*_*m*_ values of PEDV, TGEV, PoRV, PDCoV, PRV, and PRRSV were 78.12°C, 83.25°C, 80.09°C, 81.64°C, 85.83°C, and 84.03°C, respectively, with differences of more than 1°C between each other, enabling clear differentiation.

### 3.3. Specificity and *T*_*m*_ Values of Multiplex HRM Real‐Time PCR

**Figure 3:**
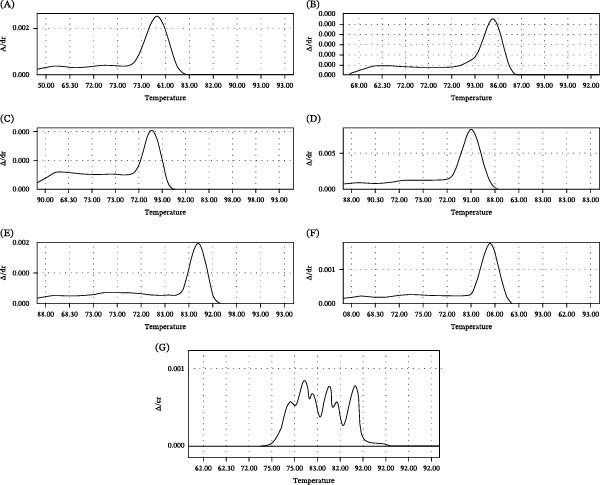
Melting curves of the multiplex HRM real‐time PCR assay. (A) PEDV; (B) TGEV; (C) PoRV; (D) PDCoV; (E) PRV; (F) PRRSV; (G) mixed six viruses.

The optimized multiplex HRM real‐time PCR assay successfully amplified six viral genes, generating six distinct melting peaks (Figure 3). The *T*_*m*_ established by the multiplex assay were 78.24°C for PEDV, 83.19°C for TGEV, 80.17°C for PoRV, 81.52°C for PDCoV, 85.66°C for PRV, and 84.11°C for PRRSV, all consistent with the corresponding single‐plex assays (differences < 0.5°C). No nonspecific melting peaks were detected in the negative control, and the *T*_*m*_ values of the mixed viruses were identical to those of the individual viruses.

### 3.4. Specificity Analysis

**Figure 4:**
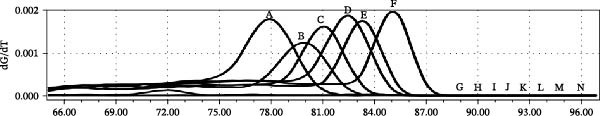
Specificity melting curves of the multiplex HRM real‐time PCR assay. (A) PEDV; (B) PoRV; (C) PDCoV; (D) TGEV; (E) PRRSV; (F) PRV; (G) CSFV; (H) PCV2; (I) PCV3; (J) PPV; (K) JEV; (L) negative control; (M) healthy porcine tissue; (N) fecal matrix samples.

**Figure 5:**
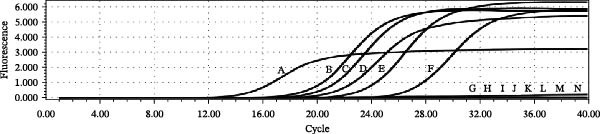
Specificity amplification curves of the multiplex HRM real‐time PCR assay. (A) TGEV; (B) PRV; (C) PDCoV; (D) PEDV; (E) PRRSV; (F) PoRV; (G) CSFV; (H) PCV2; (I) PCV3; (J) PPV; (K) JEV; (L) negative control; (M) healthy porcine tissue; (N) fecal matrix samples.

The multiplex HRM real‐time PCR assay specifically amplified the six target viruses, producing distinct melting peaks. No cross‐reactivity was observed with CSFV, PCV2, PCV3, PPV, or JEV (Figures 4 and 5), indicating high specificity.

The optimized multiplex HRM real‐time PCR assay specifically amplified the six target viruses, with each target producing a distinct and single melting peak. No cross‐reactivity was observed with nontarget porcine pathogens (CSFV, PCV2, PCV3, PPV, and JEV), and no nonspecific amplification or melting peaks were detected in healthy porcine tissue and fecal matrix samples. No amplification signal was observed in the NTC. These results confirm that the established assay has excellent specificity without nonspecific amplification caused by pathogen cross‐reaction or sample matrix interference.

### 3.5. Sensitivity and Standard Curves

**Figure 6:**
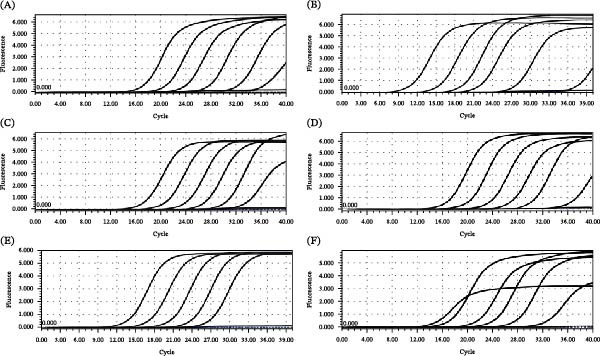
Amplification curve of the multiplex HRM quantitative PCR detection method. The amplification curve from left to right corresponds to 1 × 10^6^ to 1 × 10^0^ copies/μL. (A) PEDV; (B) TGEV; (C) PoRV; (D) PDCoV; (E) PRV; (F) PRRSV.

**Figure 7:**
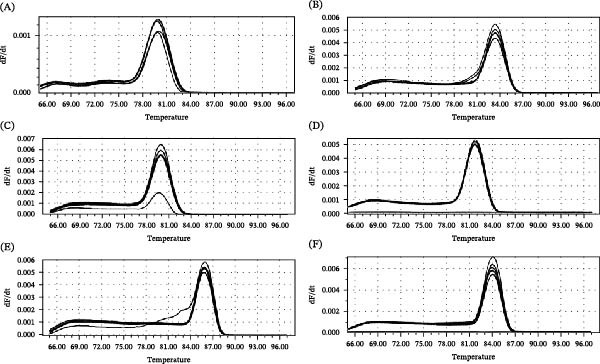
Melting curve of the multiplex HRM fluorescence quantitative PCR detection method. (A) PEDV; (B) TGEV; (C) PoRV; (D) PDCoV; (E) PRV; (F) PRRSV.

The multiplex assay demonstrated LODs of 10 copies/μL for PEDV, TGEV, PoRV, PDCoV, and PRRSV and 100 copies/μL for PRV (Figure 6). Melting curve analysis of PEDV, TGEV, PoRV, PDCoV, PRV, and PRRSV revealed that each of the six pathogens displayed a single, distinct melting peak without nonspecific multipeak artifacts across all the tested concentration gradients. This observation confirms the high specificity of the developed multiplex HRM real‐time PCR assay. Specifically, the *T_m values_* of the standard plasmid samples for PEDV, TGEV, PoRV, PDCoV, PRV, and PRRSV were determined as follows: 78.24 ± 0.15°C, 83.27 ± 0.12°C, 80.11 ± 0.13°C, 81.62 ± 0.19°C, 85.81 ± 0.11°C, and 84.01 ± 0.16°C (Figure 7).

**Figure 8:**
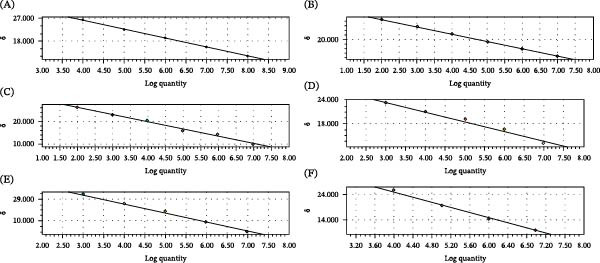
Standard curves of the multiplex HRM real‐time PCR assay. (A) PEDV; (B) TGEV; (C) PoRV; (D) PDCoV; (E) PRV; (F) PRRSV.

The standard curves showed excellent linearity in the range from 1 × 10^6^ to 1 × 10^2^ copies/μL, with *R* ^2^ values ≥ 0.98 and amplification efficiencies ranging from 90% to 102% (Table 3 and Figure 8).

**Table 3:** Standard curve parameters of the multiplex HRM real‐time PCR assay.

Virus	Regression equation	*R* ^2^	Amplification efficiency (%)
PEDV	*Y* = −3.3537*X* + 35.56	1.00	99
TGEV	*Y* = −3.2706*X* + 33.03	0.99	102
PoRV	*Y* = −3.4370*X* + 33.96	0.98	95
PDCoV	*Y* = −3.330*X* + 35.63	1.00	100
PRV	*Y* = −3.6220*X* + 36.33	1.00	90
PRRSV	*Y* = −3.3537*X* + 40.26	1.00	92

The intra‐assay CVs of the *C*_*t*_ and *T*_*m*_ values were <3% and <0.5%, respectively. The interassay CVs of the *C*_*t*_ and *T*_*m*_ values were also <3% and <0.5%, indicating excellent reproducibility of the assay (Tables 4 and 5).

**Table 4:** Intra‐assay repeatability of the multiplex HRM real‐time PCR assay (*C*_*t*_ values).

Target	Intra‐assay				Interassay		
	Plasmid concentration (copies/µL)	*C*_*t*_ mean	SD	CV (%)	*C*_*t*_ mean	SD	CV (%)
PEDV	1 × 10^6^	15.68	0.11	0.70	15.93	0.06	0.38
	1 × 10^4^	22.39	0.21	0.94	22.54	0.24	1.06
	1 × 10^2^	30.70	0.64	2.08	31.15	0.81	2.60
TGEV	1 × 10^6^	14.51	0.26	1.79	15.12	0.07	0.46
	1 × 10^4^	22.02	0.19	0.86	22.37	0.31	1.39
	1 × 10^2^	36.67	0.59	1.61	35.63	0.73	2.05
PoRV	1 × 10^6^	15.64	0.13	0.83	15.72	0.14	0.89
	1 × 10^4^	22.33	0.31	1.39	23.13	0.16	0.69
	1 × 10^2^	28.86	0.53	1.84	29.71	0.78	2.63
PDCoV	1 × 10^6^	15.46	0.32	2.07	15.54	0.33	2.12
	1 × 10^4^	22.13	0.58	2.62	22.43	0.37	1.65
	1 × 10^2^	28.85	0.48	1.66	29.14	0.62	2.13
PRV	1 × 10^6^	13.88	0.21	1.51	14.16	0.31	2.19
	1 × 10^4^	20.77	0.32	1.54	21.25	0.41	1.93
	1 × 10^2^	26.13	0.27	1.03	27.11	0.29	1.07
PRRSV	1 × 10^6^	14.21	0.13	0.91	14.13	0.25	1.77
	1 × 10^4^	20.41	0.12	0.59	20.15	0.45	2.23
	1 × 10^2^	26.52	0.54	2.04	27.69	0.74	2.67

**Table 5:** Interassay repeatability of the multiplex HRM real‐time PCR assay (*T*_*m*_ values).

Target	Intra‐assay				Interassay		
	Plasmid concentration (copies/µL)	*C*_*t*_ mean	SD	CV (%)	*C*_*t*_ mean	SD	CV (%)
PEDV	1 × 10^6^	78.18	0.12	0.15	78.28	0.15	0.19
	1 × 10^4^	78.22	0.16	0.20	78.28	0.25	0.32
	1 × 10^2^	78.18	0.11	0.14	78.28	0.21	0.27
TGEV	1 × 10^6^	83.25	0.12	0.14	83.28	0.18	0.22
	1 × 10^4^	83.30	0.19	0.23	83.35	0.21	0.25
	1 × 10^2^	83.27	0.23	0.28	83.36	0.24	0.29
PoRV	1 × 10^6^	80.11	0.15	0.19	80.13	0.28	0.35
	1 × 10^4^	80.13	0.19	0.24	80.19	0.24	0.30
	1 × 10^2^	80.07	0.23	0.29	80.16	0.27	0.34
PDCoV	1 × 10^6^	81.66	0.18	0.22	81.59	0.32	0.39
	1 × 10^4^	81.57	0.21	0.26	81.69	0.23	0.28
	1 × 10^2^	81.62	0.15	0.18	81.55	0.26	0.32
PRV	1 × 10^6^	85.74	0.17	0.20	85.89	0.30	0.35
	1 × 10^4^	85.87	0.13	0.15	85.54	0.22	0.26
	1 × 10^2^	85.59	0.13	0.15	85.67	0.28	0.33
PRRSV	1 × 10^6^	84.01	0.16	0.19	84.07	0.31	0.37
	1 × 10^4^	84.03	0.12	0.14	84.02	0.15	0.18
	1 × 10^2^	84.17	0.24	0.29	84.11	0.25	0.30

### 3.6. Clinical Sample Detection

Clinical evaluation results demonstrated that the detection rates of PEDV, TGEV, PoRV, PDCoV, PRV, and PRRSV using the multiplex HRM real‐time PCR assay were 89.5%, 88.0%, 90.9%, 92.5%, 95.5%, and 91.4%, respectively, which were 23.7%, 16.0%, 27.3%, 22.1%, 24.4%, and 17.1% higher than those obtained by conventional PCR (Table 6). The overall detection rate of the multiplex assay was 18.1% higher than that of the conventional PCR. No false positives were detected among 45 negative samples, further confirming the assay’s high specificity.

**Table 6:** Comparison of detection rates between multiplex HRM real‐time PCR and conventional PCR.

Virus	Number of samples	Multiplex HRM real‐time PCR		Conventional PCR	
		Positive number	Detection rate (%)	Positive number	Detection rate (%)
PEDV	38	34	89.5	25	65.8
TGEV	25	22	88.0	18	72.0
PoRV	33	30	90.9	21	63.6
PDCoV	27	25	92.5	19	70.4
PRV	45	43	95.5	32	71.1
PRRSV	35	32	91.4	26	74.3
Negative samples	45	0	0.0	0	0.0

Gold‐standard validation via bidirectional Sanger sequencing was performed on all HRM‐positive clinical samples with a cycle threshold (*C*_*t*_) value <28, covering all six target viruses. The sequencing results confirmed 100% concordance with the typing results of our developed HRM assay.

Analysis of 248 field samples revealed a substantial 29.9% (74/248) mixed infection rate, comprising dual (19.4%), triple (8.5%), and quadruple (2.0%) infections (Table 7). The prevailing PEDV + PoRV pairing (41.9%) parallels 2025 national surveillance, reporting high comorbidity between these enteropathogens. PRRSV‐associated clusters accounted for 32.4% of cases, echoing its known role as an immunosuppressive pathogen prone to polymicrobial infection.

**Table 7:** Statistics of mixed infection of six swine viruses in 248 clinical samples.

Infection type	Number of positive samples	Positive rate in total samples (%)	Proportion in all positive samples (%)	Dominant coinfection combination
Single infection	174	70.1	70.1	—
Dual infection	48	19.4	19.4	PEDV + PoRV
Triple infection	21	8.5	8.5	PRRSV + PEDV + PoRV
Quadruple infection	5	2.0	2.0	PEDV + TGEV + PoRV + PDCoV
Total mixed infection	74	29.9	29.9	—

## 4. Discussion

The diagnosis for porcine viral diseases has shifted from single pathogen detection to the management of complex syndromic coinfections. This approach not only meets clinical needs but also significantly improves the efficiency of disease detection or diagnosis. The simultaneous prevalence of PEDV, TGEV, PoRV, PDCoV, PRV, and PRRSV poses a persistent challenge to global swine biosecurity. Notably, PEDV continues to be a leading cause of neonatal mortality, even with widespread vaccination. Recent epidemiological surveys in China emphasize that mixed infections have become commonplace in intensive farming systems, highlighting an urgent need for multiplex diagnostic solutions [21, 22]. In this study, we successfully developed a multiplex HRM‐based real‐time PCR assay that provides a robust, high‐throughput solution to this diagnostic challenge.

A major challenge in multiplex detection of multiple viral pathogens is the potential for primer interference and template competition, in which the high viral load of one pathogen may inhibit the amplification of coinfecting agents present at lower concentrations. To mitigate this issue, the reaction system was systematically optimized by adjusting primer concentrations, balancing the ratios among different virus‐specific primers, and refining amplification parameters such as the annealing temperature. Furthermore, all primers were designed to target highly conserved genomic regions to ensure stable amplification efficiency and maintain diagnostic accuracy despite the genetic diversity and ongoing evolution of these viruses [23].

Our results, showing consistent amplification efficiencies (90%–102%) and clear *T*_*m*_ separation, suggest that our assay can accurately differentiate variants even amid the genetic plasticity of recent years. For example, despite the emergence of novel recombinant PRRSV strains in Southern China [24], our primers, designed against stable genomic footprints, maintained broad reactivity. Furthermore, the use of HRM for feline calicivirus differentiation has previously proven effective for wild‐type and vaccine strain discrimination, reinforcing the reliability of melting curve analysis in clinical settings [25].

Regarding analytical performance, our assay demonstrated a sensitivity of 10–100 copies/μL, which is comparable to the precision of digital PCR (dPCR) platforms recently applied to enteric coronaviruses [26]. However, while dPCR provides absolute quantification, its prohibitive cost and specialized equipment requirements limit its utility for routine large‐scale surveillance. In contrast, our HRM‐based method eliminates the need for expensive fluorophore‐labeled TaqMan probes [27], offering a “probe‐free” differentiation approach that significantly reduces the economic burden on producers. This economic viability is critical given the increasing cost of veterinary diagnostics in the global market [28]. Moreover, the closed‐tube format also minimizes the risk of laboratory‐acquired aerosol contamination, a persistent issue in high‐volume testing facilities [29].

Initial feasibility trials revealed that integrating a porcine internal control exacerbated primer interference and melt peak congestion, impeding the detection of low‐copy templates. Adhering to established precedents for high‐order veterinary multiplexing [30–32], we opted to maximize the amplification efficiency and *T*_*m*_ resolution by foregoing the seventh target. Procedural reliability was maintained through stringent negative extraction controls, NTCs, and positive plasmid standards. Assay validity hinged on unambiguous positive amplification alongside quiescent NTC signals; any anomalies prompted immediate retesting to preclude false negatives.

The observed 29.9% mixed infection rate exemplifies the burgeoning complexity of swine disease landscapes in China. The prevalence of PEDV/PoRV coinfections aligns closely with field reports, approaching 90% in diarrheal samples. Moreover, frequent PRRSV identification—often exceeding 84% in multipathogen contexts—corroborates its role as the primary immunosuppressive driver of clinical coinfections. Full concordance with Sanger sequencing establishes the HRM‐based multiplex assay’s bench‐top reliability. Unlike conventional single‐plex assays, this closed‐tube method simultaneously profiles six major viruses, optimizing surveillance throughput and reducing costs for early intervention in high‐density production environments. This enhanced sensitivity is crucial for early intervention as the synergistic effects of enteric and respiratory coinfections can drastically increase piglet mortality. While recent advances in point‐of‐care testing (POCT) emphasize on‐farm diagnostics [33], the laboratory‐based multiplex HRM remains the “gold standard” for comprehensive epidemiological surveillance and strain tracking.

Despite its strengths, this study is subject to several limitations. First, clinical validation was performed using samples collected from a limited number of provinces in China. Owing to the continuous genetic evolution of these viruses and the global emergence of novel viral lineages, ongoing surveillance and periodic reassessment of primer–template complementarity are necessary to maintain diagnostic accuracy. Second, the assay necessitates a real‐time PCR platform capable of HRM analysis. Although such instruments are commonly available in well‐equipped clinical and research laboratories, this dependency could constrain the assay’s applicability in certain resource‐limited veterinary diagnostic settings. Nonetheless, the growing accessibility of compact and economical real‐time PCR platforms is progressively facilitating the adoption of HRM‐based diagnostics. Finally, since HRM discrimination relies on differences in melting temperature (*T*_*m*_) profiles generated after PCR amplification, direct integration of this approach into purely isothermal amplification systems presents a significant technical challenge. Future studies may therefore aim to adapt the assay for use with portable real‐time PCR platforms, microfluidic PCR systems, or other miniaturized molecular diagnostic devices. Additionally, broadening the detection panel to include emerging pathogens such as African swine fever virus (ASFV) would further improve the platform’s utility and support integrated pathogen surveillance within a broader One Health framework [34].

## 5. Conclusion

In this study, a multiplex HRM‐based real‐time PCR assay was successfully developed for the simultaneous detection and differentiation of PEDV, TGEV, PoRV, PDCoV, PRV, and PRRSV. The assay exhibited high specificity, sensitivity, and reproducibility, with a detection limit of 10–100 copies/μL, an amplification efficiency of 90%–102%, and CVs <3% for *C*_*t*_ values and <0.5% for *T*_*m*_ values. Clinical evaluation revealed that the assay had a higher detection rate than conventional PCR did, making it suitable for the rapid diagnosis and epidemiological surveillance of mixed infections caused by these six major porcine viral pathogens. This assay provides a powerful technical tool for the effective control of swine viral diseases and promotes the healthy development of the global swine industry.

## Author Contributions

**Fangshan Wang**: writing – original draft, writing – review and editing, data curation, formal analysis, methodology, investigation. **Jing Ren**: formal analysis, methodology, data curation, writing – original draft, writing – review and editing. **Yawen Wang**: formal analysis, methodology, investigation. **Chen Yuan**: data curation, formal analysis, investigation. **T** **airan Sun**: investigation, resources, data curation. **Jinhai Huang**: resources, sample collection, investigation. **Qinye Song**: conceptualization, supervision, project administration, funding acquisition, methodology, writing – review and editing.

## Acknowledgments

The authors are grateful for the financial support from the Shijiazhuang Municipal Government and Hebei Province Natural Science Foundation Committee, China.

## Funding

This work was funded by the Enterprise‐University‐Research Cooperation Project for Hebei‐based Universities of Shijiazhuang Municipal Government (Grant 241500417A) and the Joint Special Project of Natural Science Foundation of Beijing‐Tianjin‐Hebei Region (Grant 25JJJJC0018).

## Disclosure

All authors read and approved the final manuscript.

## Ethics Statement

The sample collection was approved by the Experimental Animal Management and Ethics Committee of Hebei Agricultural University (Approval Number 2023171). Clinical samples used in this study were collected as part of routine disease surveillance and diagnostic investigations in pig farms. Sample collection was performed by trained veterinarians and did not involve any additional procedures beyond standard veterinary practice.

## Conflicts of Interest

The authors declare no conflicts of interest.

## Data Availability

The datasets used in the current study are available from the corresponding author upon reasonable request.
